# Amplification of 3q26.2, 5q14.3, 8q24.3, 8q22.3, and 14q32.33 Are Possible Common Genetic Alterations in Oral Cancer Patients

**DOI:** 10.3389/fonc.2020.00683

**Published:** 2020-04-30

**Authors:** Melvin A. Ambele, Andre van Zyl, Michael S. Pepper, Marlene B. van Heerden, Willie F. P. van Heerden

**Affiliations:** ^1^Department of Oral Pathology and Oral Biology, Faculty of Health Sciences, School of Dentistry, University of Pretoria, Pretoria, South Africa; ^2^Department of Immunology, and SAMRC Extramural Unit for Stem Cell Research and Therapy, Faculty of Health Sciences, Institute for Cellular and Molecular Medicine, University of Pretoria, Pretoria, South Africa; ^3^Specialist in Oral Medicine and Periodontics, Private Practice, Stellenbosch, South Africa

**Keywords:** oral squamous cell carcinomas, head and neck cancer, genomic heterogeneity, intratumor clonal heterogeneity, intertumoral clonal diversity, OncoScan® FFPE assay, VELscope®Vx device

## Abstract

The lack of clinical biomarkers for head and neck cancer subtypes limits early diagnosis and monitoring of disease progression. This study investigates genetic alterations in clinically identical tumor, tumor-adjacent dysplastic epithelium (TADE) and normal epithelium (NE) in five oral cancer patients to identify differences and commonalities between oral cancer, TADE and NE. A VELscope®Vx device was used to identify TADE and NE surrounding a clinical tumor for analysis of genetic alterations using the OncoScan® assay. One of the tumor samples examined was an “M” class tumor with a high confidence *BRAF*:p.G469A:c.1406G>C somatic mutation, which is the first to be reported in oral cancer. Another tumor showed mosaicism in genetic alterations, indicating the presence of multiple clones. Overall, each patient's tumor, TADE and NE showed a distinct genetic profile which indicates intertumoral clonal/genetic diversity. Interestingly, four tumors showed gain of 3q26.2, 5q14.3, 8q24.3, 8q22.3, 14q32.33 and loss/LOH in 9p21.3 while all TADE had LOH on 22q11.23. In addition, some genetic alterations progressed from NE through TADE into tumor in individual patients. Furthermore, no molecular event was identified that is common to all NE and/or TADE that progressed into tumor. This pilot study demonstrates the presence of genetic heterogeneity in oral tumorigenesis, and suggests that there might exist some common genetic alterations between tumors and TADE. However, this observation would need to be further investigated and validated in a larger cohort of oral cancer patients for its potential role in oral tumorigenesis.

## Introduction

The Globocan 2018 statistics reported 887,659 new cases of head and neck cancer (lip & oral cavity; 354,864, salivary gland; 52,799, oropharynx; 92,887, nasopharynx; 129,079, hypopharynx; 80,608 and larynx; 177,422 cases) with 453,307 deaths worldwide (http://gco.iarc.fr/today/fact-sheets-cancers). Significant genomic instability and resultant clonal diversity are hallmark characteristics of head and neck squamous cell carcinoma (HNSCC). Heterogeneity in phenotype, etiology, biology and clinical presentation are common features of HNSCC, and this may account for the dismal 5-year survival rates which are as low as 50% in all patients, despite treatment. Except for tumor human papilloma virus (HPV) status, the molecular risk factors investigated in HNSCC have yielded limited clinical utility. Risk stratification for HNSCC is based largely on the tumor anatomical site, stage and histological characteristics (1). Of interest is oral squamous cell carcinoma (OSCC) that develops through a multistep process involving the accumulation of multiple genetic mutations. This process is influenced both by genetic predisposition and environmental risk factors such as tobacco, alcohol, and HPV infection (2). Differences in tumor evolution and progression as well as resistance to therapy in oral cancer can be attributed to intratumoral clonal heterogeneity and intertumoralal clonal diversity, which are seen in clinical cases of oral cancer and other cancers of the head and neck region (1, 3–6). Understanding the molecular mechanisms of clonal heterogeneity within a tumor and clonal diversity between tumors may lead to better treatment outcomes in affected patients.

Field cancerization is to date the most acceptable molecular progression model for OSCC development. The model was proposed by Slaughter et al. and linked the presence of dysplastic changes in tumor-adjacent epithelium of oral cancer specimens with local recurrence and multifocal areas of cancer development process in many cells as a result of exposure to a carcinogen such as tobacco (7). A previous study in our group has also demonstrated this concept of field cancerization in OSCC with heterozygosity in p16 expression (8). Genetic alterations, mainly loss of heterozygosity (LOH) or deletion in chromosomal regions 9p (*CDKN2A*) (2, 9), 3p (*FHIT* & *RSSFIA*) (10, 11), and 17p (*TP53*) (2) have been observed in relatively high proportions of dysplastic lesions and are considered early events in oral carcinogenesis. Losses at 13q and 8p are observed more frequently in carcinomas than in dysplasia, and are associated with late stages of oral carcinogenesis (2, 12).

Inactivation of tumor suppressor genes (TSGs) by loss of heterozygosity (LOH) or deletion and/or the activation of oncogenes by gene amplification are the two major types of genetic alterations most often associated with OSCC tumorigenesis (1, 12, 13). In this study, a VELscope®Vx Handpiece (LED Dental Inc, Canada) was used to identify tumor-adjacent dysplastic epithelium (TADE) and normal epithelium (NE) surrounding a clinically detected cancer. The Affymetrix OncoScan® FFPE assay was then used to investigate the genetic alterations in these specimens with the aim of identifying common molecular events in all tumor, TADE and NE samples. Significant findings could be further explored in a larger cohort of oral cancer patients for their potential role in early to late stages of oral tumorigenesis.

## Materials and Methods

### Clinical Diagnosis of Oral Cancer Patients

Patients with OSSC were referred to the Pretoria Oral and Dental Hospital. Each patient was seen in a specialist clinic where the primary lesion was identified, and a scalpel biopsy was done for histological confirmation of the OSSC. Written informed consent was obtained from all five patients and the study was approved by the Faculty of Health Sciences Research Ethics Committee (Reference number 44/2010).

### VELscope® Screening of Oral Cancer Patients and Tissue Collection

**Figure 1:**
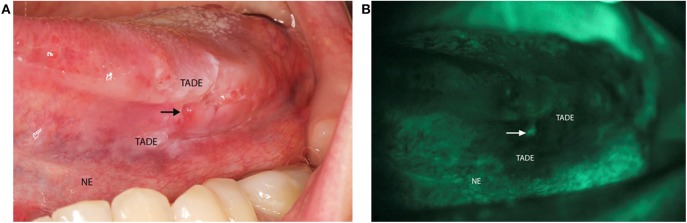
Clinical photo of VELscope® oral cancer screening of a patient. **(A)** Image of a patient with a squamous cell carcinoma on the left side of the tongue presenting as an ulcer (arrow). The tumor adjacent dysplastic epithelium (TADE) that showed loss of fluorescence with the VELscope® is present around the ulcer with the distant clinical normal epithelium (NE) that did not show loss of fluorescence. **(B)** Photograph of the VELscope® analysis showing the ulcer (arrow) with surrounding TADE with loss of autofluorescence and NE showing normal autofluorescence. (Number of patients screened = 5).

The VELscope®Vx Handpiece (LED Dental Inc, Canada) is a portable device with a 40–460 nm light source that has been shown to be effective at identifying lesions at risk of developing cancer in clinically normal mucosa (14). This device has a sensitivity of 98% and a specificity of 100% for identification of oral dysplasia and cancer (15). VELscope® is a diagnostic aid that when applied, results in pale green autofluorescence of normal mucosa and shows loss of fluorescence with abnormal tissue. This fluorescence is viewed through the narrow-band filter built into the eyepiece (16). It has also been shown to be effective at identifying lesions at risk of developing cancer in clinically normal mucosa as seen under normal light (14). One study showed that VELscope® is able to identify LOH at 3p and 9p in mucosa surrounding a clinically detected cancer, with margins as wide as 25 mm (17). Hence, each patient received a comprehensive intra-oral clinical examination with the use of the VELscope® to identify TADE and NE sites (Figure 1) for tissue sampling.

### DNA Extraction, Quantification, and OncoScan® FFPE Assay

**Figure 2:**
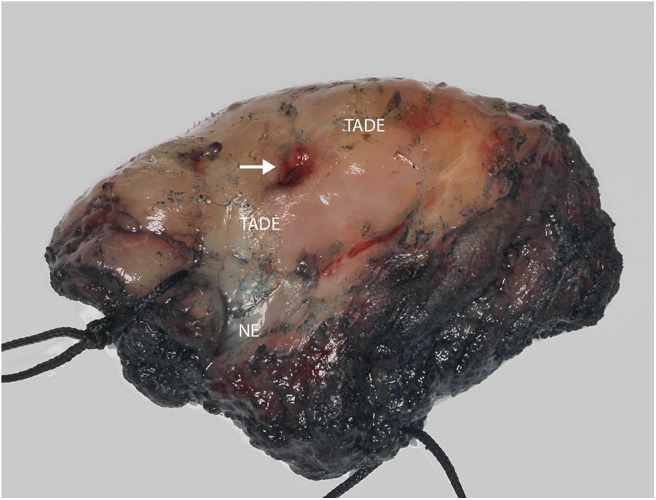
Photograph of hemi-glossectomy specimen. Image showing the carcinoma (arrow) as well as the TADE and NE areas (as determined previously using the VELscope®, number of patients screened = 5).

**Figure 3:**
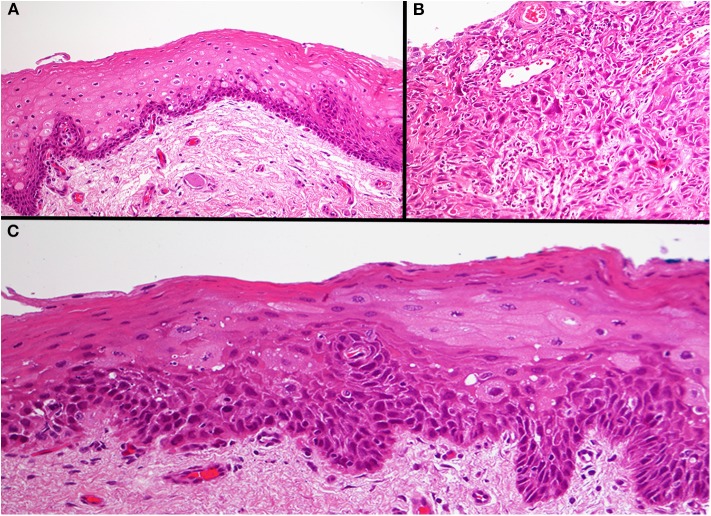
Photomicrograph of the three different areas. Histology of the normal epithelium showing basal cell hyperplasia with no atypia **(A)**. The base of the tumor ulcer consisted of a poorly differentiated squamous cell carcinoma **(B)**. The tumor adjacent dysplastic epithelium (TADE) that showed loss of fluorescence with the VELscope® showed the presence of moderate epithelial dysplasia **(C)**. Original magnification X200.

Formalin fixed paraffin embedded (FFPE) specimens of tumor, TADE and NE were prepared from hemi-glossectomy specimens (Figure 2) with different histological classification (Figure 3). Genomic DNA (gDNA) was extracted from each FFPE sample (tumor, TADE and NE) using the QIAamp DNA FFPE Tissue kit (QIAGEN GmbH, Hilden, Germany) according to the manufacturer's protocol. DNA was quantified using a Quant-iT™ PicoGreen® dsDNA Assay Kit (Life Technologies) following the manufacturer's recommended protocol. Eighty nanogram gDNA was prepared for each sample and run on the Affymetrix OncoScan® FFPE assay kit (Affymetrix; Thermo Fisher Scientific company) according to the manufacturers' instructions (18). The Affymetrix OncoScan® FFPE assay is a molecular analytical tool that works efficiently on formalin-fixed paraffin-embedded (FFPE) samples. It provides a comprehensive coverage of whole genome copy number alterations, LOH and somatic mutations of genes that have been implicated in cancer and tumor progression by utilizing the molecular inversion Probe (MIP) technology (18). Briefly, to each 80 ng of gDNA sample, copy number variation and somatic mutations MIP mixes was added followed by denaturation for 5 min at 95°C and an overnight annealing for 17 h at 58°C. The product of each sample was split into two wells to which dATP (A) and dTTP (T) (A/T) was added into one well while dGTP (G) and dCTP (C) (G/C) was added to the other well to perform a gap fill reaction according to the manufacture's manual. A cocktail of exonucleases supplied with the OncoScan® kit was used to digest gDNA and uncircularized MIP. Cleavage enzymes supplied with the OncoScan® kit were then used to linearize the circular MIP that had been gap filled by A/T or G/C nucleotides followed by a PCR amplification. A second round of PCR amplification was then performed using Haeii enzyme supplied with the OncoScan® kit to cleave amplicons from the previous PCR reaction. The cleaved fragments were then hybridized onto the OncoScan® assay array overnight for 17 h according the manufacturer's protocol. The arrays were then washed and stained using the Affymetrix GeneChip® Fluidics Station 450 and scanned on the Affymetrix GeneChip® Scanner 3000 7G. Each scanned array generates array images known as a DAT file that were automatically converted into fluorescence intensity (CEL) files by the Affymetrix® GeneChip® Command Console® (AGCC) software version 4. CEL files were imported onto the Affymetrix OncoScan^TM^ Console software version 1.3 and processed to generate OSCHP files which were analyzed using Chromosome Analysis Suite (ChAS) for copy number alterations (CNAs) and LOH in approximately 900 cancer genes as well as for 74 clinically actionable somatic mutations in nine cancer genes (*BRAF, EGFR, IDH1, IDH2, KRAS, NRAS, PIK3CA, PTEN*, and *TP53*) (18).

## Results

### Sample Size and Classification

Fifteen clinical specimens consisting of 5 tumors, 5 TADE and 5 distant NE from five oral cancer patients were examined in this pilot study for genetic changes such as copy number alterations (CNAs), loss of heterozygosity (LOH) and other somatic mutations (SMs). Of the five tumor samples, four were characterized by high CNAs (referred to as C class tumors) and showed a progressive development of genomic instability characterized by LOH and CNAs from NE through TADE to tumor. One patient's tumor was characterized by a high confidence somatic mutation with few CNAs (referred to as an M class tumor) (19) when compared to the TADE and NE in the same patient.

### Genetic Alterations Reveal Both Intratumoral Clonal Heterogeneity and Intertumoral Clonal Diversity

Tumor 1 showed a characteristic HNSCC mutation profile including the amplification of 3q26/28 (*TP63, SOX2, PIK3CA*), *FGFR1*, 11q13 (*CCND1, FADD, CTTN*), *E2F1, PTK2*, loss of *NSD1* and LOH in *CDKN2A* and *TP53* (20). The TADE and NE showed mutations in certain chromosomal regions that do not contain any of the well described HNSCC associated gene mutations. However, some observed molecular events such as the size of a Cn-LOH on 5q14.3 in NE, increased in TADE and changed to a CNL-LOH in the tumor. Also, Cn-LOH on 16q21 in NE and TADE changed to a CNL-LOH in the tumor. Extensive functional studies would be important to validate the significance of these two events in the development and progression of this tumor.

Tumor 2 is an “M” class tumor (tumor driven by mutation rather than CNAs) typified by a high confidence *BRAF*:p.G469A:c.1406G>C somatic mutation with less genomic instability compared to the TADE and NE. This somatic mutation resulted from the substitution of glycine (G) by alanine (A) at position 469 in *BRAF*. This mutation has been reported in non-small-cell lung cancer (21–23) and in colorectal cancer (24) but not in oral cancer. Loss in *FHIT* and *RB1* were the only well described HNSCC associated mutations (25) detected in TADE and which were absent from both the tumor and NE, suggesting this TADE is an independent clone and not related to the tumor. Interestingly, the size of a loss on 3p26.3 in NE that increased in this TADE would be important to investigate further for possible role in early genetic event in the NE, since a loss in this region has been suggested to be an independent prognostic factor in OSCC patients (26, 27).

Tumor 3 showed mosaicism in CNAs which is indicative of a highly heterogenous tumor with multiple clones. This tumor showed mosaicism in the amplification of *TP63, PIK3CA, SOX2, FADD, CTTN, CCND1, NOTCH1, E2F1, HRAS, BIRC2, EGFR, MYC, PTK2*, loss in *FHIT* and *CDKN2A*, and LOH in *NSD1, FAT1* and *APC*. All these mutations constitute a combination of distinct mutation profiles for various molecular sub-types of HNSCC of different etiology (1, 13, 25). Genetic alterations in TADE and NE did not involve any well described HNSCC associated mutations.

Tumor 4 showed amplification of *PIKC3A, SOX2, TP63, EGFR, PTK2, NOTCH 1, 2 & 3, AJUBA, TRAF3, ERBB2, MYC*, and *KMT2D*, loss of *CDKN2A, FAT1, APC, RB1, KLK12, SMAD4*, and *PTEN*, and LOH of *FHIT, CDKN2A, CASP8, TP53* and *NFE2L2*, which have all been reported in HNSCC (1, 13). TADE and NE had no detectable HNSCC associated mutations. However, the size of a LOH at 3p21.31 in NE and TADE increased in the tumor. In addition, the size of a homozygous deletion of 19p13.2 in NE and TADE that increased in the tumor; this warrants further investigation as it may have played a role in oral cancer development and progression in this patient.

Tumor 5 had a high confidence *PIK3CA*:p.H1047R:c.3140A>G somatic mutation resulting from the substitution of histidine (H) by arginine (R) at position 1047 of the *PIK3CA* gene. This somatic mutation has previously been detected in HNSCC patients (28). This tumor also showed additional mutations such as amplification of *TP63, SOX2, PIK3CA, FADD, CTTN, CCND1, EGFR, FGFR1, CASP8, SMAD4, TP53, NFE2L2, NOTCH1, 2,& 3, MYC, PTK2, AJUBA, TRAF3, ERBB2, NRAS, KRAS, HRAS, FAT1, KEAP1, E2F1*, and *SMAD2*, loss of *RASSF1, FHIT, CDKN2A, KMT2D, RB1, APC, CSMD1, PTPRD, MET, CUL3*, and *NSD1* and a LOH of *TP53, NOTCH1, APC, RB1, CSMD1, PTPRD, CUL3, NSD1, CDKN2A, FHIT*, and *RASSF1*, which have been reported in HNCSS (1, 13, 25). Mutations detected in TADE and NE were not those that have been well described to be associated with HNSCC.

### Identification of Molecular Events Common to Histologically Identical Samples From all Patients

We examined the mutation profile of the three groups of histologically identical sample types (tumor, TADE, and NE) from all five patients in an attempt to identify genetic alterations that are unique to each sample in the different groups (inter-patient heterogeneity) as well as those that are common to all samples in each group, which could potentially be explored in a larger cohort study of oral cancer patients for their clinical significance.

#### Tumor

With the exception of tumor 2, all tumor samples showed characteristic mutations of HNSCC including amplification of 3q26.2 (*TP63, SOX2, PIK3CA*) (1), 5q14.3 (*APC*) (13, 29, 30), 8q24.3 (*PTK2*) (25), 8q22.3 (*LRP12*) (27) as well as loss and/or LOH of 9p21.3 (*CDKN2A*) (1, 31). Other characteristic HNSCC mutations such as loss of 4q35.1 (*FAT1*) (1, 31), 5q35.2 (*NSD1*) (1, 31), LOH of 17p13.2 (*TP53*) (1, 31), and amplification of 7p11.2 (*EGFR*) (1, 31), 8q24.21 (*MYC*) (1, 25, 32) and 11q13.3 (*CCND1, FADD, CTTN*) (1) were detected in three tumors. Interestingly, we also found genetic alterations not previously described in HNSCC such as amplification of 14q32.33 (*LINC00221*) and 20q11.22 in 4 tumors, and loss and/or LOH of 5q23.2, 5q35.1 and loss of 3p24.1 (*NEK10*) in three tumors. There were many other undescribed detectable genetic alterations present in at least 3 tumor samples (Table S1). Despite some common molecular features of HNSCC detected in all tumor samples, the type of alteration (gain, loss or LOH) detected in some of the affected chromosomal locations and in certain HNSCC associated genes, differ from one tumor to another, thereby making each tumor genetically distinct (Table 1).

**Table 1:** Genetic alterations on the same chromosomal location common to at least three tumors.

**Chromosome cytoband start location**	**Types of alteration in tumor**				
	**Tumor 1**	**Tumor 2**	**Tumor 3**	**Tumor 4**	**Tumor 5**
1q31.1	–	LOH	–	Gain	Gain
3p26.3 (26, 27)	–	–	Loss	Loss	Loss
4q31.2	–	–	Loss	Gain	Gain
4q35.1 (*FAT1*) (1, 31)	–	–	Loss	Loss	Gain
5q11.2	Loss	–	–	Loss	Loss
6p25.2	LOH	–	Gain	Gain	LOH
7p11.2 (*EGFR*) (1, 31)	–	–	Gain	Gain	Gain
9p23	LOH	Gain	Gain	Loss	LOH
11q13.3 (*CCND1, FADD, CTTN*) (1)	Gain	–	Gain	–	Gain
12q24.12 (*ALDH2*)	LOH	LOH	–	Gain	–
13q13.1	Loss	–	–	LOH	Loss
15q11.2	Gain	–	Loss	Loss	Gain
16p13.3	LOH	–	Loss	Gain	Gain
17p13.2 (*TP53*) (1, 31)	LOH	–	–	LOH	Gain + LOH
20q11.21	Gain	–	Gain	–	Gain
22q11.23 (*GSTT1*)	–	–	Gain	Gain	Gain

*The same chromosomal location revealed intertumoral genetic diversity of all five tumors based on either (a) a discrepancy in the genetic alterations (gain, loss, or LOH) or (b) the presence/absence of a genetic alteration only in certain tumors*.

#### Tumor-Adjacent Dysplastic Epithelium (TADE)

Except for TADE 2, all TADE showed fewer CNAs compared to their respective tumor samples. The number of genetic alterations that were common to at least 3 TADE were fewer and different from those found among tumor samples (Table 2). Interestingly, a LOH event on 22q11.23 (*GSTTP1, LOC391322, GSTT1, GSTTP2*) was present in all 5 TADE. Other alterations detected in at least 3 TADE includes amplification of 1p13.3 (*GSTM2, GSTM1, GSTM5*), 14q32.33 (*LINC00221*) and LOH of 3p21.1 (*NEK4*) and 3p21.31. *NEK4* has not been reported in HNSCC tumorigenesis and this gene is known to regulate cell entry into replicative senescence as well as the response to double strand DNA damage (33). Thus, LOH of *NEK4* in OSCC should be functionally investigated further as it could be suggestive of a putative TSG in the development of dysplastic lesions in the oral mucosal. Genetic alterations of a specific type (gain, loss or LOH) in affected chromosomal locations/genes were consistent among TADE unlike in tumor samples. Notwithstanding the limited number of mutations found to be common among TADE, there were alterations that were present and/or absent in a subset of TADE thereby resulting in each patient's TADE having a unique genetic profile (Table 2).

**Table 2:** Genetic alterations on the same chromosomal location common to at least two TADE.

**Chromosome cytoband start location**	**Types of alteration in TADE**				
	**TADE 1**	**TADE 2**	**TADE 3**	**TADE 4**	**TADE 5**
1p13.3 (*GSTM1*) (46)	–	Gain	–	Gain	Gain
3p21.1 (*NEK4*)	LOH	LOH	–	–	LOH
3p21.31 (*RASSF1*)	LOH	Loss	–	LOH	–
14q32.33 (*LINC00221*)	Gain	Gain	–	–	Gain
22q11.23 (*GSTT1*) (47)	LOH	LOH	Gain + LOH	LOH	Gain +LOH

*All 5 TADE showed differences in genetic alteration (LOH, gain, gain + LOH, or none) at the same loci*.

#### Normal Epithelium (NE)

In all patients, NE had fewer CNAs compared to their respective TADE. Only two genetic alterations were common to at least two out of the five NE (Table 3). Surprisingly, one of the genetic events—loss and/or LOH of 3p21.3—was common in all the five NE. Inactivating mutations in this region have been a consistent finding in cancers, especially HNSCC, and have been associated with early development of dysplastic lesions in HNSCC (13, 27, 34–43). The clinical significance of this genetic event in NE is unknown and warrants further investigation.

**Table 3:** Genetic alterations common to at least two NE.

**Chromosome cytoband start location**	**Types of alteration in NE**				
	**NE 1**	**NE 2**	**NE 3**	**NE 4**	**NE 5**
3p21.3	LOH	Loss + LOH	LOH	Loss + LOH	LOH
5q23.3	–	LOH	LOH	–	–
11p11.12	LOH	–	–	–	Gain

*Genetic alterations detected were mostly loss and LOH on chromosomes 3p, 5q, and 11p which could be used to distinguish between individual patient's NE*.

### Is There a Marker for Progression in Oral Cancer Tumorigenesis?

We examined genetic alterations present in NE and/or TADE that were also found in tumor and as such could denote progression through clonal expansion from a single progenitor clone. Such progressive genetic alterations from NE and/or TADE to tumor could be suggestive of an early marker for oral cancer development and/or progression. We could not find a single genetic event that was common to all five NE and/or TADE which progressed to tumor. This demonstrates a high level of genetic diversity among all five patients' samples. Nevertheless, the activating mutation of the novel putative oncogene *LINC00221* (14q32.33) and the inactivating mutation of the novel putative TSG *NEK4* (3p21.1) that were detected in TADE1 & 5, were retained as they progressed into their respective tumors.

## Discussion

Genetic heterogeneity in HNSCC has been described in tumors from various anatomical sites analyzed using different techniques (3, 5, 44). This pilot study reports analysis of the genome of tumor, TADE and NE from five patients with OSCC with the aim of identifying molecular events in tumor, TADE and NE samples, which could be explored further in a larger cohort study of oral cancer patients for their significance in oral tumorigenesis. Tumor from each patient showed a unique interpatient mutation profile. Differences in the types of genetic alterations (gain, loss and/or LOH) at the same chromosomal location and in some HNSCC associated genes observed in tumor samples are indicative of intertumoral genetic diversity. Tumor 2 showed a high confidence *BRAF*:p.G469A:c.1406G>C somatic mutation (classified as “M” class tumor), which is the first to be reported in oral cancer. This tumor sample showed less genomic instability compared to TADE and NE. “M” class tumors have previously been observed in oral cancer (1, 19). Except for tumor 2, all tumors were characterized by high CNAs classified as “C” class tumors. Tumor 3 showed mosaicism in CNAs, which is indicative of intratumoral clonal heterogeneity. Lack of uniformity in the mutational landscape of all 5 tumors suggests intertumoral clonal/genetic diversity. Except for tumor 2, all tumors showed focal deletion of 3p and amplification of 5p and 8q which contain genes not previously described to be associated with HNSCC. Furthermore, amplification of 14q32.33 (*LINC00221*) and 20q11.22 in all 4 tumors and a loss and/or LOH of 5q23.2, 5q35.1 and 3p24.1 (*NEK10*) in three tumors are interesting findings which would require more detailed functional studies to evaluate their possible role as putative oncogenes and TSGs, respectively, in these regions. *NEK10*, not previously described in HNSCC tumorigenesis, has been reported to mediate G2/M cell cycle arrest (45) and could be a potential TSG in oral cancer.

Interestingly, TADE from all 5 patients showed inactivating mutations of 22q11.23 (*GSTTP1, LOC391322, GSTT1, GSTTP2*). The clinical significance of this genetic alteration in all five patients is unknown. However, given the presence of a traditional risk factor (tobacco consumption) in all patients, further studies are warranted.

Surprisingly, all five NE showed inactivating mutations of 3p21.3, known to contain TSGs or resident cancer genes. Alterations in this region are one of the most consistent genetic events reported not only in HNSCC but also in other cancer types (13, 27, 34–43). The presence of this genetic alteration in NE of all patients is therefore very surprising; could it possibly be that this particular region of the human genome is naturally very unstable? Further investigation of NE in both healthy individuals and oral cancer patients would be beneficial in understanding this genomic region and its associated genetic alterations.

## Conclusion

Amplification of 3q26.2, 5q14.3, 8q24.3, 8q22.3, 14q32.33, 20q11.22 together with a loss/LOH on 9p21.3 was detected in four out of the five oral cancer samples, and a LOH on 22q11.23 detected in all five TADE. Furthermore, no molecular event was identified that is common to all NE and/or TADE that progressed into tumor. The small sample size limits the clinical significance of these findings. We therefore recommend further studies in a larger cohort of oral cancer patients to determine their significance in oral cancer biology.

## Data Availability Statement

The datasets generated for this study are available on request to the corresponding author.

## Ethics Statement

The studies involving human participants were reviewed and approved by University of Pretoria Faculty of Health Sciences Research Ethics Committee (Reference number 44/2010). The patients/participants provided their written informed consent to participate in this study.

## Author Contributions

MA performed the OncoScan® assay, data analysis, interpretation, and wrote the first draft of the manuscript. AZ conceptualized the study, performed clinical examination of patients, the Velscope® screening, data interpretation, and edited the manuscript, MP contributed to the study design, data interpretation, edited the manuscript, and provided funding for the study. MH prepared FFPE specimen of all tissues, performed DNA isolation, and edited the manuscript. WH conceptualized the study, performed clinical examination of patients, data interpretation, edited the manuscript, and provided funding for the study.

## Conflict of Interest

The authors declare that the research was conducted in the absence of any commercial or financial relationships that could be construed as a potential conflict of interest.
