# Supplementary material for: Amplification of 3q26.2, 5q14.3, 8q24.3, 8q22.3, and 14q32.33 Are Possible Common Genetic Alterations in Oral Cancer Patients
Source: Front Oncol. 2020 Apr 30;10:683. doi: 10.3389/fonc.2020.00683 (PMC7203479; doi:10.3389/fonc.2020.00683)
Supplement: Supplementary file 1 [file Table_1.DOCX]

**Table S1:** List of genetic alterations detected in at least three tumor samples. Tumor samples show high degree of intertumoral heterogeneity based on the presence/absence of genetic alteration as well as the discrepancy in the type of alterations (gain, loss or LOH) on the same chromosomal location.

| Chromosome number | Tumor 1 (Female) | Tumor 2 (Male) | Tumor 3 (Male) | Tumor 4 (Male) | Tumor 5 (Male) | Comments |
| --- | --- | --- | --- | --- | --- | --- |
| Chr1 |  |  | LOH at 1p22.2 | Gain at 1p22.2 | Gain at 1p22.2 |  |
|  |  |  | LOH at 1q25.3 | Gain at 1q25.3 | Gain at 1q25.3 |  |
|  |  | LOH at 1q31.1 |  | Gain at 1q31.1 | Gain at 1q31.1 |  |
| Chr3 |  |  | Loss at 3p26.3 + LOH | Loss at 3p26.3 + LOH | loss at 3p26.3 + LOH | Loss in this region has been suggested to be an independent prognostic factor in OSCC patients[1, 2] |
|  |  |  | Loss at 3p25.1 | Loss at 3p25.1 | Loss at 3p25.1 |  |
|  | LOH at 3p24.3 |  | Loss at 3p24.3 |  | Loss at 3p24.3 |  |
|  |  |  | Loss at 3p24.1 (*NEK10*) | Loss at 3p24.1 (*NEK10*) | Loss at 3p24.1 (*NEK10*) | *NEK10* is a gene that mediates G2/M cell cycle arrest[3] which has not been implicated or reported in HNSCC |
|  | LOH at 3p21.31 |  | Loss at 3p21.31 | LOH at 3p21.31 | Loss at 3p21.31 |  |
|  | LOH at 3p21.1 (*NEK4*) |  | Loss + LOH at 3p21.1 (*NEK4*) | Loss + LOH at 3p21.1 (*NEK4*) | Loss + LOH at 3p21.1 (*NEK4*) | NEK4 regulates entry into replicative senescence and response to dsDNA damage[4], thus loss of function mutation could lead to limitless replicative potential and propagation of mutation in DNA |
|  |  |  | Loss at 3p13 | Loss at 3p13 | Loss at 3p13 |  |
|  |  |  | Gain at 3q11.2 | Gain at 3q11.2 | Gain at 3q11.2 |  |
|  |  |  | Gain at 3q13.31 | Gain at 3q13.31 | Gain at 3q13.31 |  |
|  |  |  | Gain at 3q13.3 | Gain at 3q13.3 | Gain at 3q13.3 |  |
|  | Gain at 3q22.3 |  | Gain at 3q22.3 | Gain at 3q22.3 | Gain at 3q22.3 |  |
|  | Gain at 3q26.2 (*TP63*, *SOX2*, *PIK3CA*) |  | Gain at 3q26.2 (*TP63*, *SOX2*, *PIK3CA*) | Gain at 3q26.2 (*TP63*, *SOX2*, *PIK3CA*) | Gain at 3q26.2 (*TP63*, *SOX2*, *PIK3CA*) | Gain in this region is a characteristic feature of HNSCC[5] |
| Chr4 |  |  | Gain at 4q28.1 | Loss at 4q28.1 | Gain at 4q28.1 |  |
|  |  |  | Loss at 4q28.1 | Loss at 4q28.1 | Gain at 4q28.1 |  |
|  |  |  | Loss at 4q31.2 | Gain at 4q31.2 | Gain at 4q31.2 |  |
|  |  |  | Loss at 4q35.1 (*FAT1*) | Loss at 4q35.1 (*FAT1*) | Gain at 4q35.1 (*FAT1*) | Inactivating mutation of *FAT1* has been reported in HNSCC[5, 6], hence tumor 5 is different from tumor 3 & 4 |
| Chr5 | Gain at 5p15.33 |  |  | Gain at 5p15.33 | Gain at 5p15.33 | Gain of 5p is typical in HNSCC[5] |
|  | Gain at 5p15.2 |  | Gain at 5p15.2 | Gain at 5p15.2 | Gain at 5p15.2 |  |
|  | Gain at 5p15.1 |  | Gain at 5p15.1 | Gain at 5p15.1 | Gain at 5p15.1 |  |
|  | Loss at 5q11.1 |  |  | Loss at 5q11.1 | Loss at 5q11.1 |  |
|  | Loss at 5q11.2 |  |  | Loss at 5q11.2 | Loss at 5q11.2 |  |
|  | Loss at 5q14.3 (*APC*) |  | LOH at 5q14.3 (*APC*) | Loss at 5q14.3 (*APC*) | Loss at 5q14.3 (*APC*) | LOH in *APC* has been reported in OSCC[7-9] |
|  | Loss at 5q23.2 |  | LOH at 5q23.2 | Loss at 5q23.2 | Loss at 5q23.2 | None |
|  | Loss at 5q23.1 |  | LOH at 5q23.1 |  | Loss at 5q23.1 |  |
|  | Loss at 5q35.1 |  | LOH at 5q35.1 | Loss at 5q35.1 | Loss at 5q35.1 |  |
|  | Loss at 5q35.2 (*NSD1*) |  | LOH at 5q35.2 (*NSD1*) |  | Loss at 5q35.2 (*NSD1*) | Inactivation of *NSD1* reported in HNSCC[5, 6] |
|  |  |  |  |  |  |  |
| Chr6 |  |  | Gain at 6p25.3 | Gain at 6p25.3 | Loss at 6p25.3 |  |
|  | LOH at 6p25.2 |  | Gain at 6p25.2 | Gain at 6p25.2 | LOH at 6p25.2 |  |
|  |  |  | Gain at p22.1 | Gain at p22.1 | LOH at p22.1 |  |
|  | Gain at 6p12.1 |  |  | Gain at 6p12.1 | Gain at 6p12.1 |  |
|  |  |  | Gain at 6p11.2 | Gain at 6p11.2 | Gain at 6p11.2 |  |
|  |  |  | Gain at 6q11.1 | Gain at 6q11.1 | Gain at 6q11.1 |  |
|  |  |  | Loss at 6q26 | Gain at 6q26 | Gain at 6q26 |  |
| Chr7 |  |  | Gain at 7p22.3 | Gain at 7p22.3 | Gain at 7p22.3 |  |
|  |  |  | Gain at 7p15.2 | Gain at 7p15.2 | Loss at 7p15.2 |  |
|  |  |  | Gain at 7p15.2 | Gain at 7p15.2 | Gain at 7p15.2 |  |
|  |  |  | Gain at 7p14.2 | Loss at 7p14.2 | Gain at 7p14.2 |  |
|  |  |  | Gain at 7p11.2 (*EGFR*) | Gain at 7p11.2 (*EGFR*) | Gain at 7p11.2 (*EGFR*) | *EGFR* amplification is common in HNSCC[5, 6] |
|  |  | Somatic mutation on *BRAF* | LOH at 7q34 (*BRAF*) | Gain at 7q34 (*BRAF*) | Loss at 7q34 (*BRAF*) | *BRA*F mutation reported in OSCC but at a very low frequency[10-12] |
| Chr8 | Gain at 8q11.1 |  | Gain at 8q11.1 | Gain at 8q11.1 | Gain at 8q11.1 | Frequent amplification of 8q (*LRP12*) is common to OSCC[2]  *MYC* and *FGFR1* amplification implicated in HNSCC[5, 13, 14] |
|  | Gain at 8q12.1 |  | Gain at 8q12.1 | Gain at 8q12.1 | Gain at 8q12.1 |  |
|  | Gain at 8q12.3 |  | Gain at 8q12.3 | Gain at 8q12.3 | Gain at 8q12.3 |  |
|  | Gain at 8q13.3 |  | Gain at 8q13.3 | Gain at 8q13.3 | Gain at 8q13.3 |  |
|  | Gain at 8q21.3 |  | Gain at 8q21.3 | Gain at 8q21.3 | Gain at 8q21.3 |  |
|  | Gain at 8q22.2 |  | Gain at 8q22.2 | Gain at 8q22.2 | Gain at 8q22.2 |  |
|  | Gain at 8q22.3 (*LRP12*) |  | Gain at 8q22.3 (*LRP12*) | Gain at 8q22.3 (*LRP12*) | Gain at 8q22.3 (*LRP12*) |  |
|  | Gain at 8q23.3 |  | Gain at 8q23.3 | Gain at 8q23.3 | Gain at 8q23.3 |  |
|  |  |  | Gain at 8q24.21 (*MYC*) | Gain at 8q24.21 (*MYC*) | Gain at 8q24.21 (*MYC*) |  |
|  | Gain at 8q24.23 |  | Gain at 8q24.23 | Gain at 8q24.23 | Gain at 8q24.23 |  |
|  | Gain at 8q24.3 (*PTK2*) |  | Gain at 8q24.3 (*PTK2*) | Gain at 8q24.3 (*PTK2*) | Gain at 8q24.3 (*PTK2*) | *PTK2* is candidate oncogene in HNSCC[13] |
| Chr9 | LOH at 9p24.3 |  | Gain at 9p24.3 | LOH at 9p24.3 | Loss at 9p24.3 |  |
|  | LOH at 9p23 | Gain at 9p23 | Gain at 9p23 | LOH at 9p23 | loss at 9p23 |  |
|  | LOH at 9p21.3 (*CDKN2A*) |  | Loss at 9p21.3 (*CDKN2A*) | Loss at 9p21.3 (*CDKN2A*) | Loss at 9p21.3 (*CDKN2A*) | Inactivating mutation of *CDKN2A* is associated with HNSCC[5, 6] |
|  | LOH at 9p21.3 |  | Gain at 9p21.3 |  | Loss at 9p21.3 |  |
|  | LOH at 9p21.1 |  | Gain at 9p21.1 | LOH at 9p21.1 | Gain at 9p21.1 |  |
|  | LOH at 9q21.13 |  | Gain at 9q21.13 | Gain at 9q21.13 | LOH at 9q21.13 |  |
|  | LOH at 9q21.33 |  | Gain at 9q21.33 | Gain at 9q21.33 | Gain at 9q21.33 |  |
|  | LOH at 9q22.33 (*NOTCH1*) |  | Gain at 9q22.33 (*NOTCH1*) | Gain at 9q22.33 (*NOTCH1*) | Gain at 9q22.33 (*NOTCH1* + LOH) | Inactivating mutation of *NOTCH1* in HNSCC[5, 6] |
| Chr11 | LOH at 11p11.12 |  | Gain at 11p11.12 |  | Gain at 11p11.12 |  |
|  | Gain at 11q12.3 |  | Gain at 11q12.3 |  | Gain at 11q12.3 |  |
|  | Gain at 11q13.3 (*CCND1*, *FADD*, *CTTN*) |  | Gain at 11q13.3 (*CCND1*, *FADD*, *CTTN*) |  | Gain at 11q13.3 (*CCND1*, *FADD*, *CTTN*) | Amplification in *CCND1*, *FADD*, *CTTN* reported in HNSCC[5] |
| Chr12 | LOH at 12q12 |  |  | Gain at 12q12 | Gain at 12q12 |  |
|  | LOH at 12q13.13 |  |  | Gain at 12q13.13 | Gain at 12q13.13 |  |
|  | LOH at 12q15 |  |  | Gain at 12q15 | Gain at 12q15 |  |
|  |  |  |  | Gain at 12q13.12 (*KMT2D*) | Loss at 12q13.12 (*KMT2D*) | *KMT2D* is frequently mutated in HNSCC[5, 6] |
|  |  | LOH at 12q24.11 | LOH at 12q24.11 | Gain at 12q24.11 |  |  |
|  | LOH at 12q24.12 (*ALDH2*) | LOH at 12q24.12 (*ALDH2*) |  | Gain at 12q24.12 (*ALDH2*) |  |  |
|  | LOH at 12q24.13 | LOH at 12q24.13 |  | Gain at 12q24.13 | Gain at 12q24.13 |  |
| Chr13 | Loss at 13q13.1 |  |  | LOH at 13q13.1 | Loss at 13q13.1 |  |
|  |  |  |  | Loss at 13q14.2 (*RB1*) | Loss at 13q14.2 (*RB1*) | Loss of *RB1* is reported in HNSCC[5, 6, 13] |
| Chr14 | Gain at 14q32.33(*LINC00221*) |  | Gain at 14q32.33 (*LINC00221*) | Gain at 14q32.33 (*LINC00221*) | Gain at 14q32.33 (*LINC00221*) |  |
| Chr15 | Gain at 15q11.2 |  | Loss at 15q11.2 | Loss at 15q11.2 | Gain at 15q11.2 |  |
|  |  |  | Loss at 15q15.1 | Loss at 15q15.1 | Gain at 15q15.1 |  |
| Chr16 | LOH at 16p13.3 |  | Loss at 16p13.3 | Loss at 16p13.3 | Gain at 16p13.3 |  |
|  | LOH at 16p13.3 |  | Loss at 16p13.3 | Gain at 16p13.3 | Gain at 16p13.3 |  |
|  | Loss at 16p11.2 |  | Loss at 16p11.2 | Gain at 16p11.2 |  |  |
|  |  | LOH at 16p11.2 | Gain (+LOH) at 16p11.2 | Gain (+LOH) at 16p11.2 | Gain at 16p11.2 |  |
|  |  |  | Gain at 16q12.1 | Gain at 16q12.1 | Gain at 16q12.1 |  |
|  | Loss at 16q21 |  | Gain at 16q21 | Gain at 16q21 | Gain at 16q21 |  |
|  | LOH at 16q22.1 |  | Gain at 16q22.1 | Loss at 16q22.1 | Gain at 16q22.1 |  |
|  |  |  | Gain at 16q22.2 | Gain at 16q22.2 | Gain at 16q22.2 |  |
|  |  |  | Gain at 16q23.1 | Gain at 16q23.1 | Gain at 16q23.1 |  |
| Chr17 | LOH at 17p13.3 |  |  | Gain at 17p13.3 | Gain at 17p13.3 |  |
|  | LOH at 17p13.2 (*TP53*) |  |  | LOH at 17p13.2 (*TP53*) | Gain (+LOH) at 17p13.2 (*TP53*) | Inactivating mutation of *TP53* associated HNSCC[5, 6, 13] |
|  | Gain (+LOH) at 17p13.1 |  |  | LOH at 17p13.1 | Gain (+LOH) at 17p13.1 |  |
|  |  |  |  | Gain at 17p11.2 | Loss at 17p11.2 |  |
|  |  |  |  | Loss at 17q12 | Gain t 17q12 |  |
|  |  |  |  | Gain at 17q12 (*ERBB2*) | Gain (+LOH) at 17q12 (*ERBB2*) | Amplification of *ERBB2* reported in HNSCC[5] |
|  |  |  |  | Gain at 17q21.1 | Loss at 17q21.1 |  |
| Chr20 |  |  | Gain at 20p13 | Gain at 20p13 | Gain at 20p13 |  |
|  |  |  | Gain at 20p12.1 | Loss at 20p12.1 | Gain at 20p12.1 |  |
|  |  |  | Gain at 20p11.21 | Gain at 20p11.21 | Gain at 20p11.21 |  |
|  | Gain at 20q11.21 |  | Gain at 20q11.21 |  | Gain at 20q11.21 |  |
|  | Gain at 20q11.22 |  | Gain at 20q11.22 | Gain at 20q11.22 | Gain at 20q11.22 |  |
|  |  |  | Gain at 20q13.2 | Gain at 20q13.2 | Gain at 20q13.2 |  |
| Chr21 |  |  | Loss at 21p11.2 | Loss at 21p11.2 |  |  |
|  |  |  | LOH at 21q21.1 | LOH at 21q21.1 | LOH at 21q21.1 |  |
|  |  |  | Loss at 21q21.3 | LOH at 21q21.3 | LOH at 21q21.3 |  |
|  |  |  | Loss at 21q22.12 | Gain at 21q22.12 | LOH at 21q22.12 |  |
| Chr22 |  |  | Gain at 22q11.23 (*GSTTP1*, *LOC391322*, *GSTT1*, *GSTTP2*) | Gain at 22q11.23 (*GSTTP1*) | Gain at 22q11.23 (*GSTTP1*, *LOC391322*, *GSTT1*, *GSTTP2*) |  |
| ChrX | Gain at Xp22.33 |  | Loss at Xp22.33 | Gain at Xp22.33 | Gain at Xp22.33 |  |
|  | Gain at Xq11.1 |  | Gain at Xq11.1 | Gain at Xq11.1 |  |  |
|  |  |  | Gain at Xq12 (*AR*) | Gain at Xq12 (*AR*) | Gain at Xq12 (*AR*) |  |
|  |  |  | Gain at Xq21.1 | Gain at Xq21.1 | Gain at Xq21.1 |  |
|  |  |  | Gain at Xq24 | Gain at Xq24 | Gain at Xq24 |  |
|  |  |  | Gain at Xq27.3 | Gain at Xq27.3 | Gain at Xq27.3 |  |

**References**

[1] Uchida K, Oga A, Nakao M, Mano T, Mihara M, Kawauchi S, et al. Loss of 3p26.3 is an independent prognostic factor in patients with oral squamous cell carcinoma. Oncol Rep. 2011;26:463-9.

[2] Vincent-Chong VK, Salahshourifar I, Woo KM, Anwar A, Razali R, Gudimella R, et al. Genome wide profiling in oral squamous cell carcinoma identifies a four genetic marker signature of prognostic significance. PloS one. 2017;12:e0174865.

[3] Moniz LS, Stambolic V. Nek10 mediates G2/M cell cycle arrest and MEK autoactivation in response to UV irradiation. Mol Cell Biol. 2011;31:30-42.

[4] Nguyen CL, Possemato R, Bauerlein EL, Xie A, Scully R, Hahn WC. Nek4 regulates entry into replicative senescence and the response to DNA damage in human fibroblasts. Mol Cell Biol. 2012;32:3963-77.

[5] The Cancer Genome Atlas N, Lawrence MS, Sougnez C, Lichtenstein L, Cibulskis K, Lander E, et al. Comprehensive genomic characterization of head and neck squamous cell carcinomas. Nature. 2015;517:576.

[6] Leemans CR, Snijders PJF, Brakenhoff RH. The molecular landscape of head and neck cancer. Nature reviews Cancer. 2018;18:269-82.

[7] Largey JS, Meltzer SJ, Sauk JJ, Hebert CA, Archibald DW. Loss of heterozygosity involving the APC gene in oral squamous cell carcinomas. Oral surgery, oral medicine, and oral pathology. 1994;77:260-3.

[8] Rivero ER, Horta MC, Silva Guerra EN, Ferraz AR, Nunes FD. Loss of heterozygosity of the APC gene in oral squamous cell carcinoma. Pathology, research and practice. 2008;204:793-7.

[9] Kasamatsu A, Uzawa K, Usukura K, Koike K, Nakashima D, Ishigami T, et al. Loss of heterozygosity in oral cancer. Oral Science International. 2011;8:37-43.

[10] Bruckman KC, Schönleben F, Qiu W, Woo VL, Su GH. Mutational analyses of the BRAF, KRAS, and PIK3CA genes in oral squamous cell carcinoma. Oral surgery, oral medicine, oral pathology, oral radiology, and endodontics. 2010;110:632-7.

[11] Chen F, Zhang Q, Wang Y, Wang S, Feng S, Qi L, et al. KIT, NRAS, BRAF and FMNL2 mutations in oral mucosal melanoma and a systematic review of the literature. Oncology Letters. 2018;15:9786-92.

[12] Soma PF, Pettinato A, Agnone AM, Donia C, Improta G, Fraggetta F. Oral malignant melanoma: A report of two cases with BRAF molecular analysis. Oncology Letters. 2014;8:1283-6.

[13] Leemans CR, Braakhuis BJ, Brakenhoff RH. The molecular biology of head and neck cancer. Nature reviews Cancer. 2011;11:9-22.

[14] Rodrigo JP, Lazo PS, Ramos S, Alvarez I, Suarez C. MYC amplification in squamous cell carcinomas of the head and neck. Archives of otolaryngology--head & neck surgery. 1996;122:504-7.
